# The incubation for urethral gonorrhoea among men who have sex with men with and without oropharyngeal gonorrhoea

**DOI:** 10.1017/S095026882400089X

**Published:** 2024-09-27

**Authors:** Julien Tran, Christopher K. Fairley, Jason J. Ong, Tiffany R. Phillips, Ei T. Aung, Eric P. F. Chow

**Affiliations:** 1 Melbourne Sexual Health Centre, Alfred Health, Melbourne, VIC, Australia; 2 School of Translational Medicine, Faculty of Medicine, Nursing and Health Sciences, Monash University, Melbourne, VIC, Australia; 3 Centre for Epidemiology and Biostatistics, Melbourne School of Population and Global Health, The University of Melbourne, Melbourne, VIC, Australia

**Keywords:** gonorrhoea, incubation, MSM, sexually transmitted infection, multisite infection

## Abstract

We hypothesized that the incubation for urethral gonorrhoea would be longer for men with oropharyngeal gonorrhoea than those without oropharyngeal gonorrhoea. We conducted a chart review of men who have sex with men with urethral gonorrhoea symptoms at a sexual health clinic between 2019 and 2021. The incubation period was defined as the number of days between men’s last sexual contact and onset of symptoms. We used a Mann–Whitney *U* test to compare differences in the median incubation for urethral gonorrhoea between men with and men without oropharyngeal gonorrhoea. There were 338 men with urethral symptoms (median age = 32 years; IQR: 28–39), and of these, 307 (90.1%) were tested for oropharyngeal gonorrhoea, of whom 124 (40.4%, 95% CI: 34.9–46.1) men had oropharyngeal and urethral gonorrhoea. We analyzed incubation data available for 190 (61.9%) of the 307 men, with 38.9% (74/190) testing positive for oropharyngeal gonorrhoea. The incubation for urethral gonorrhoea did not differ between 74 men (39%) with oropharyngeal gonorrhoea (median = 4 days; IQR: 2–6) and 116 men (61%) without oropharyngeal gonorrhoea (median = 2.5 days; IQR: 1–5) (*p* = 0.092). Research is needed to investigate gonorrhoea transmission from the oropharynx to the urethra.

## Introduction

The rates of gonorrhoea are high and increasing among gay, bisexual, and other men who have sex with men (MSM) [1] in high-income countries such as Australia [2]. Condomless insertive anal sex and fellatio have been reported as common risk factors for urethral gonorrhoea among MSM [3, 4]. However, emerging research suggests that saliva may serve as a potential mode for transmitting gonorrhoea from the oropharynx to other anatomical sites [5, 6].

Research suggests that the transmission of *Neisseria gonorrhoeae* can occur through other sexual activities that involve saliva [7], such as tongue-kissing [8, 9] or using saliva as a lubricant for anal sex [10]. The possibility of transmission by auto-inoculation from saliva during masturbation was recently raised following findings from a randomized controlled trial (RCT) investigating the efficacy of antibacterial mouthwash for the prevention of gonorrhoea [11]. In this RCT, men who were allocated to use the intervention mouthwash were significantly less likely to have urethral gonorrhoea compared to those who used the control mouthwash. Furthermore, those who were diagnosed with urethral gonorrhoea were more likely to use their own saliva for masturbation compared to the overall study population [11], however, there was no conclusive evidence for the link between the use of saliva for masturbation and urethral gonorrhoea. While mathematical models have suggested that saliva use for masturbation can play an important role in the transmission of gonorrhoea [12], prospective empirical studies will be required to examine this potential route of transmission.

Most men with urethral gonorrhoea develop symptoms 2–7 days after exposure, while men with extra-genital gonorrhoea are mostly asymptomatic [13–16]. However, a previous study reported that the incubation period (i.e., the time between men’s last sexual contact and the onset of their symptoms) for urethral gonorrhoea followed a bimodal distribution, which peaked at day 4 and again at day 11 [13]. Thus, we hypothesized that the incubation period for urethral gonorrhoea may be related to the source of infection. Men with a shorter incubation period may have acquired the urethral infection directly from a partner while men with a longer incubation period may have acquired the infection first at their oropharynx and then transmitted it to their urethra by using their saliva as a lubricant for masturbation, leading to a longer time between their last sexual contact and the onset of their symptoms.

To our best knowledge, there is only one study published in 1982 that has examined factors affecting the incubation period for urethral gonorrhoea. The study by Schofield found that, on average, men with their first episode gonococcal urethritis had a longer incubation period for urethral gonorrhoea compared to men with repeated gonococcal urethritis [15]. However, Schofield did not consider factors such as the positivity of oropharyngeal gonorrhoea among men. Therefore, our study aimed to examine whether there are differences in the incubation period for urethral gonorrhoea between men with oropharyngeal gonorrhoea and men without oropharyngeal gonorrhoea.

## Methods

### Study setting and population

We conducted a retrospective chart review of medical records among MSM aged ≥16 years who attended the Melbourne Sexual Health Centre (MSHC) between July 2019 and February 2021. We defined MSM as men who have had sex with other men in the past 12 months. Upon their arrival at the MSHC, men are asked to complete questions about their demographic characteristics and sexual practices as part of their routine care through computer-assisted self-interview (CASI).

Since August 2017, all MSM attending the MSHC are offered testing for gonorrhoea at the urethra, oropharynx, and anus if they report anal sex practices regardless of symptoms [17]. First-pass urine samples are collected among men who do not have urethral symptoms, while men who have urethral symptoms undergo a genital examination and have urethral swabs taken for culture, in addition to urine samples. Anorectal and oropharyngeal swabs are also collected. Due to the COVID-19 pandemic, the clinic transitioned from clinician-collected to self-collected oropharyngeal swabs in March 2020 to minimize the risk of exposure to SARS-CoV-2 virus for healthcare workers; however, this transition did not affect the positivity rates of oropharyngeal gonorrhoea [18]. This study was approved by the Alfred Hospital Ethics Committee, Melbourne, Australia (69/21).

### Data collection

We extracted data on participants’ age, sexual contact with someone known to have gonorrhoea, as well as insertive and/or receptive anal sex, number of casual partners and condom use during sex in the past three months through CASI. We categorized men into groups based on self-reported anal sex role in the past three months with a regular and/or casual male partner. Men were ‘versatile’ if they had insertive and receptive anal sex; ‘top-only’ if they only had insertive anal sex; and ‘bottom-only’ if they only had receptive anal sex. We performed a chart review of extracted data on the number of days since men last had any sexual contact with a male partner and days since the onset of their symptoms from their medical records. This data was based on men’s recall and was recorded by clinicians at the MSHC on the day of the men’s clinic visit and during their consultation. We assumed that men’s last sexual contact was the transmitting partner and calculated the incubation period for urethral gonorrhoea by subtracting the number of days since men’s last sexual contact with a male partner and days since the onset of their urethral symptoms. For instance, for a man whose most recent sexual contact was three days ago and who develops urethral symptoms one day before his clinic visit, the incubation period for his urethral gonorrhoea would be 2 days. In our chart review, we extracted data on whether men had urethral symptoms. Typical urethral symptoms include typical discharge (yellow, green, pus-like discharge), and atypical symptoms include urethral discomfort, dysuria, or non-purulent discharge. Our study only included men with urethral symptoms on the day of their clinic visit. Oropharyngeal swabs and first-pass urine samples were tested by nucleic acid amplification test (NAAT) for *N. gonorrhoeae* using Aptima Combo 2 Assay (Hologic Panther system; Hologic, San Diego, CA). We extracted the test results for oropharyngeal gonorrhoea, chlamydia and syphilis among men who had urethral symptoms on the day of their clinic visit.

### Data analysis

We calculated the means and medians for continuous variables, the proportions and 95% confidence intervals (CI) for categorical variables using the binomial exact method. We used independent samples Kolmogorov–Smirnov test to compare differences in the distributions of incubation days between men with oropharyngeal gonorrhoea and men without oropharyngeal gonorrhoea. We performed an independent samples *t*-test to compare the mean of incubation period for urethral gonorrhoea between men with and men without oropharyngeal gonorrhoea. We also compared the mean of incubation days for urethral gonorrhoea for (1) the type of urethral symptoms (typical and atypical symptoms), (2) whether men reported sexual contact with someone known to have gonorrhoea, and (3) role in anal sex (versatile, top-only or bottom-only), as secondary outcomes. We used a one-way ANOVA to compare the mean incubation period for urethral gonorrhoea based on men’s role in anal sex. We compared the differences in median incubation period for urethral gonorrhoea between two independent groups using Mann–Whitney *U* Tests and between three or more independent groups using a Kruskal–Wallis test. All statistical analyses were conducted using Stata (Version 17, College Station, TX).

## Results

**Figure 1.**
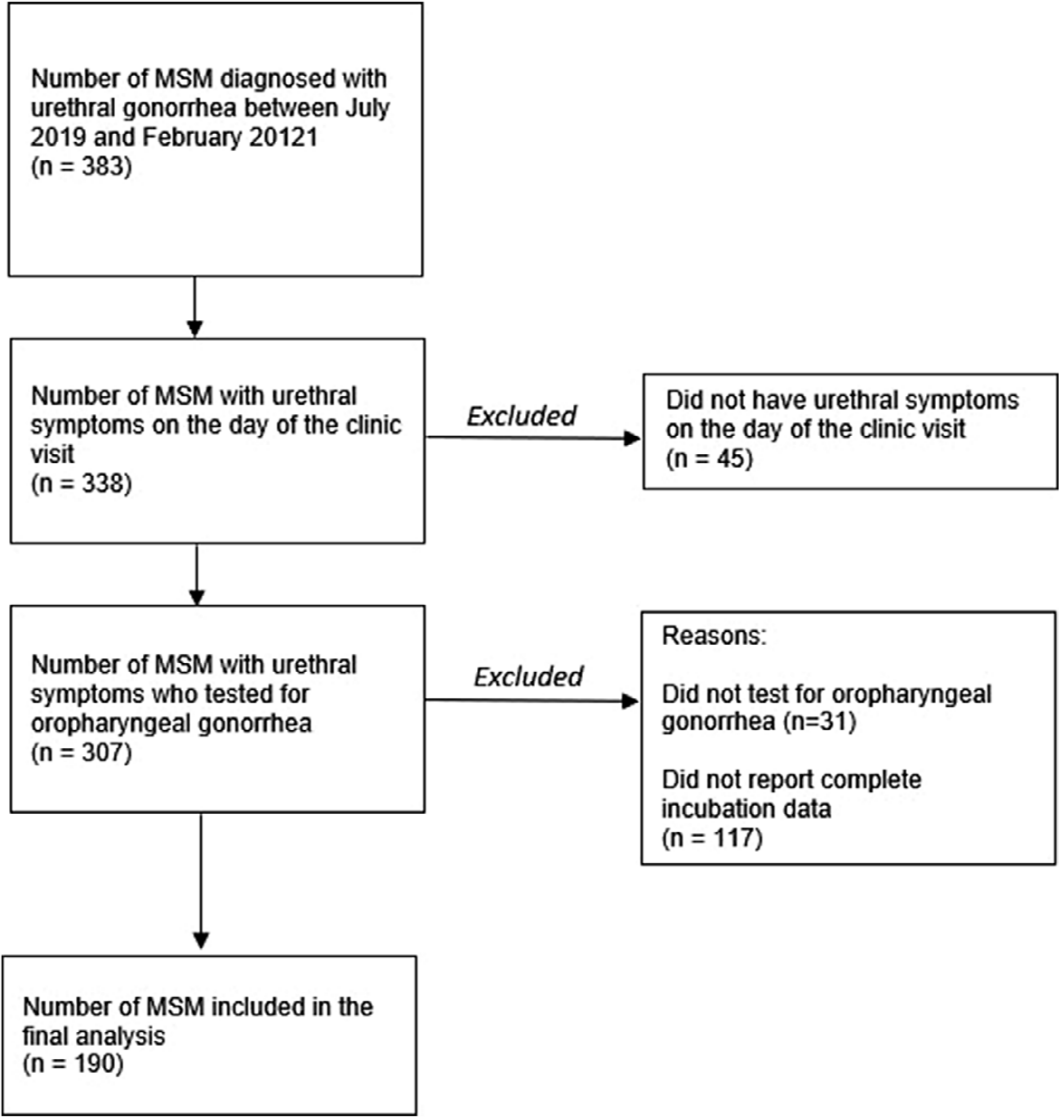
Flowchart mapping the selection process for inclusion in the final analysis. MSM, men who have sex with men.

Between July 2019 and February 2021, there were 383 men diagnosed with urethral gonorrhoea (Figure 1). There were 338 (88.3% of the 383) men who presented with urethral symptoms (median age = 32 years; IQR: 28–39), and of these men, 307 (90.1%) were tested for oropharyngeal gonorrhoea, of whom 124 (40.4%, 95% CI: 34.9–46.1) men tested positive on the same they tested positive for urethral gonorrhoea. We excluded 193 men who they did not have complete data. Of these 193 men, 45 did not report any urethral symptoms on the day of presentation, 31 were not tested for oropharyngeal gonorrhoea and 117 did not report incubation data. We only included men who had complete test results for urethral gonorrhoea and oropharyngeal gonorrhoea and incubation data in our final analysis. There were no significant differences in age (*p* = 0.715) and oropharyngeal gonorrhoea (*p* = 0.948) among men who had incubation data and men who did not have incubation data.

**Figure 2.**
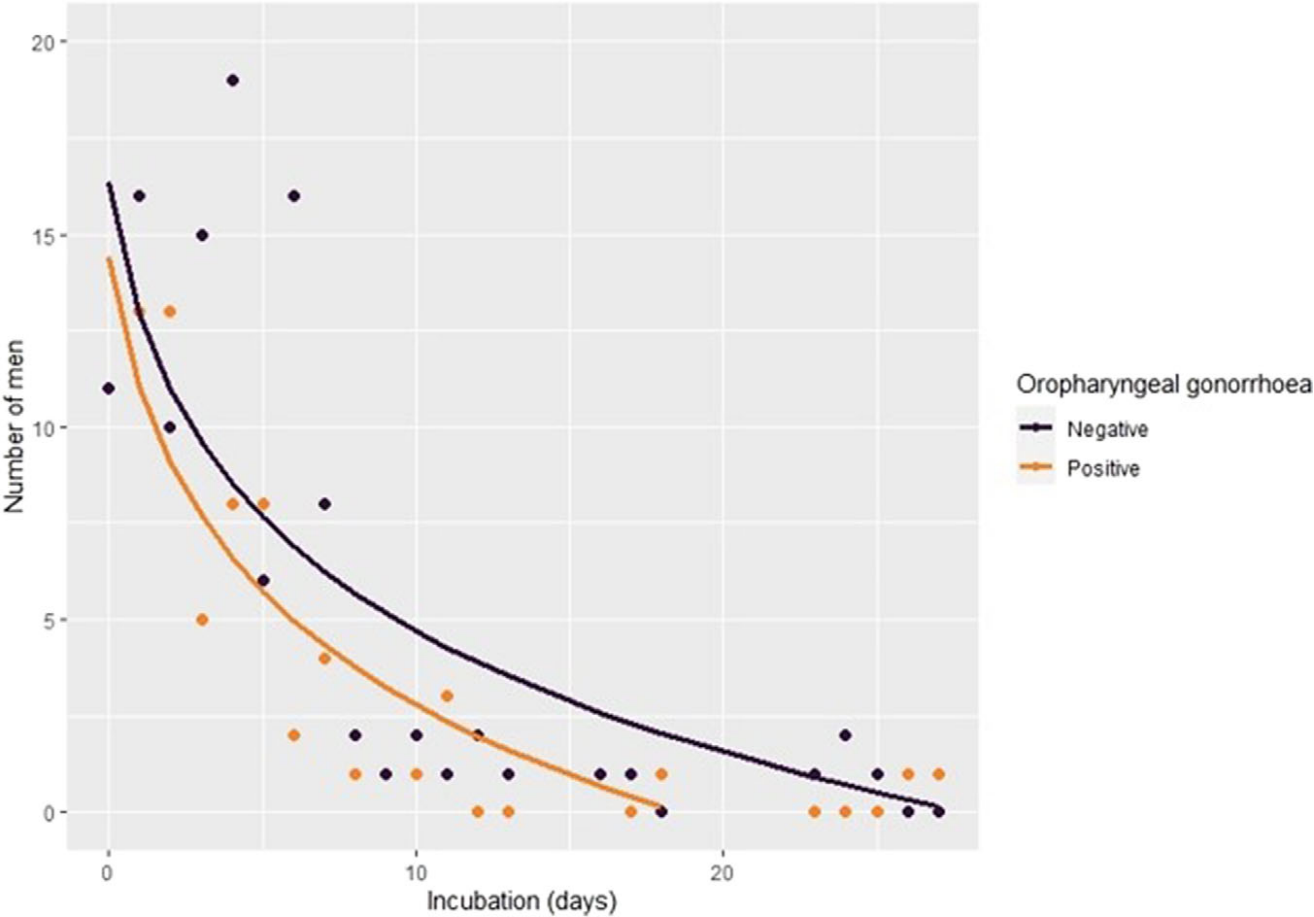
The incubation period for urethral gonorrhoea and the number of men with oropharyngeal gonorrhoea and men without oropharyngeal gonorrhoea. *Note:* The distribution of the incubation period for urethral gonorrhoea was fitted using log-transformed fractional polynomial regression.

Out of the 190 men included in the final analysis, the median age was 33 years (IQR: 28–39) and 74 (38.9%, 95% CI: 32.0–46.3) tested positive for oropharyngeal gonorrhoea. There were no significant differences in age, number of casual partners, anal sex, condom use, HIV/PrEP status, and STI co-infections between men with and men with oropharyngeal gonorrhoea (Table 1). The median incubation period for urethral gonorrhoea was 4 days (IQR:1–6). The median incubation period for urethral gonorrhoea did not significantly differ between the 74 men (2.5 days; IQR: 1–5) with oropharyngeal gonorrhoea and the 116 (4 days; IQR: 2–6) men without oropharyngeal gonorrhoea (*p* = 0.092) (Table 2). Figure 2 shows the cumulative distribution of the incubation period between these two groups. A Kolmogorov–Smirnov test showed that there were no significant differences in the cumulative distributions of incubation period between men with oropharyngeal gonorrhoea and men without oropharyngeal gonorrhoea (Figure 2, *p* = 0.103).

**Table 1.** Demographic and behavioural characteristics, and STI co-infections for 190 MSM with urethral gonorrhoea stratified by oropharyngeal gonorrhoea positivity

	Men with oropharyngeal gonorrhoea (*n* = 74)	Men without oropharyngeal gonorrhoea (*n* = 116)	*p*-value
Age			
Median (IQR)	31 (28–39)	33 (28–38)	0.493
Number of casual partners			0.976
Median (IQR)	3 (2–5)^a^	3 (2–5)^b^	
Insertive anal sex with most recent partner			0.820
Yes	48 (64.9%)	71 (61.2%)	
No	9 (20.3%)	28 (24.1)	
Not specified	5 (14.8%)	17 (14.7%)	
Condom use for anal sex^c^			0.279
Consistent	12 (16.2%)	10 (8.6%)	
Inconsistent	43 (58.1%)	74 (63.8%)	
Unknown	19 (25.7%)	32 (27.6%)	
HIV/PrEP status			0.835
Negative not on PrEP	42 (56.8%)	61 (52.6%)	
Negative on PrEP	22 (29.7%)	39 (33.6%)	
Living with HIV	10 (13.5%)	16 (13.8%)	
Chlamydia co–infection			0.515
Yes	21 (28.4%)	22 (24.1%)	
No	53 (71.6%)	88 (75.9%)	
Syphilis co–infection			0.328
Yes	3 (4.1%)	2 (1.7%)	
No	71 (95.9%)	114 (98.3%)	

*Note:* We used Mann–Whitney *U* tests to compare median age and number of casual partners, and Chi-square tests to compare differences in proportions of insertive anal sex, condom use, HIV/PrEP status, and STI co-infections between men with and men without oropharyngeal gonorrhoea.

a 31 did not report number of casual partners.

b 54 did not report number of casual partners.

c Consistent condom use: condoms used always; inconsistent condom use: condoms not always used or never used.

**Table 2.** Incubation period for urethral gonorrhoea according to the type of urethral symptoms, known gonorrhoea contact, oropharyngeal gonorrhoea positivity, and role in anal sex

	Mean (SD) incubation periods (days)	*p*-value*	Median (IQR) incubation periods (days)	*p*-value**
Oropharyngeal gonorrhoea		0.416		0.092
Positive (*n* = 74)	4.2 (5.2)		4 (2–6)	
Negative (*n* = 116)	4.8 (4.9)		2.5 (1–5)	
Urethral symptoms		0.274		0.041
Typical symptoms (*n* = 168)	4.7 (4.9)		4 (1.5–6)	
Atypical symptoms (*n* = 22)	3.5 (5.7)		2 (1–4)	
Known gonorrhoea contact***		0.565		0.563
Yes (*n* = 8)	3.6 (3.7)		2 (1.5–5.5)	
No (*n* = 181)	4.7 (5.1)		4 (1–6)	
Role in anal sex****		0.618		0.402
Versatile^a^ (*n* = 76)	5.0 (5.5)		3.5 (1–6)	
Top–only^b^ (*n* = 59)	5.1 (5.2)		4 (1–7)	
Bottom–only^c^ (*n* = 7)	3.1 (4.0)		2 (0–5)	

IQR, interquartile range; SD, standard deviation.

a Men who had insertive and receptive anal sex in the past 3 months.

b Men who only had insertive anal sex in the past 3 months.

c Men who only had receptive anal sex in the past 3 months.

* *p*-values were calculated from independent samples *t*-tests comparing differences in means.

** *p*-values were calculated using the Mann–Whitney *U* test comparing medians for two independent groups and Kruskal–Wallis test for three or more independent groups.

*** One man did not report whether he had sexual contact with someone known to have gonorrhoea.

**** Forty-eight men did not provide data for their role in anal sex.

The median incubation period for urethral gonorrhoea significantly differed between the 168 men with typical urethral symptoms (4 days; IQR: 1.5–6) and the 22 men with atypical urethral symptoms (2 days; IQR: 1–4) (*p* = 0.041) (Table 2). The median incubation period for urethral gonorrhoea did not significantly differ between the eight men who reported sexual contact with someone known to have gonorrhoea (2 days; IQR: 1.5–5.5) and the 181 men who did not report sexual contact with someone known to have gonorrhoea (4 days; IQR: 1–6) (*p* = 0.563). There were no significant differences in the median incubation period for urethral gonorrhoea among 76 men who were versatile (3.5 days; IQR: 1–6), the 59 men who were top-only (4 days; IQR: 1–7) and 7 men who were bottom-only (2 days; IQR: 0–5) (*p =* 0.0.402).

## Discussion

Only a few studies have examined the incubation period for urethral gonorrhoea in men [13–16, 19] and none of these studies has compared the incubation period for urethral gonorrhoea between men with and men without a concurrent oropharyngeal gonococcal infection. Our study found that the median incubation period for urethral gonorrhoea was 4 days, and that incubation was not significantly different between men with and men without concurrent oropharyngeal gonorrhoea. Our data do not support the hypothesis that men with a concurrent oropharyngeal gonorrhoea infection would have a longer incubation period for urethral gonorrhoea compared to those without a concurrent oropharyngeal gonorrhoea infection. Further studies will be required to explore this hypothesis by collecting data on saliva use for masturbation.

There are several possible reasons why our study did not show a difference in the incubation period for urethral gonorrhoea between men with and men without oropharyngeal gonorrhoea, if this difference did exist. First, it is possible that the bacterial load of the inoculum may influence the incubation period and the bacterial load may differ for urethral infection acquired from different sexual acts (e.g., fellatio, anal sex, and saliva use as a lubricant for masturbation) [20]. However, data on bacterial load is not available in our study because it was not collected as part of men’s routine clinical care, and future studies will be required to confirm this. Second, the actual time of infection and the person transmitting gonorrhoea are unknown. We assumed that each infection was acquired from the most recent sexual partner, but this may not have been the case, and if not, then our estimate of the incubation period would have been incorrect. Given that both urethral and oropharyngeal infections were diagnosed on the same day for some men from our study, it was possible that these infections occurred from different sexual activities during a single-sex episode [21, 22]. Due to the nature of the retrospective data, we did not have data on using saliva as a lubricant for masturbation because it is not routinely asked as part of men’s clinical care. Therefore, we were unable to draw any conclusions on the association between saliva use for masturbation and urethral gonorrhoea in our study.

Past studies have found sexual activities that involve saliva, such as kissing [8, 9, 23], performing fellatio [9, 23] and performing analingus (rimming) [9, 24] are risk factors for oropharyngeal gonorrhoea. Studies have also found that using saliva as a lubricant during anal sex is associated with anorectal gonorrhoea [10], and receiving fellatio is associated with urethral gonorrhoea [3, 4]. Many of these studies have been summarized in a review by Fairley et al. [25]. Inoculation of gonorrhoea between anatomical sites has also been reported, for example, autoinoculation from the oropharynx-to-nipple contact [26] and genital-hand-eye contact [27] in men. Given this circumstantial evidence, it is hypothesised that gonorrhoea may also be transmitted from the oropharynx to urethra by using saliva as a lubricant during masturbation. However, further studies will be required to prove this hypothesis.

Our study has several limitations not discussed above. First, this study was from a large urban sexual health clinic, and therefore, our results may not be generalizable to the broader MSM population if the men attending this service were systematically different. Second, we retrospectively collected data and not all data were available for all men attending with urethral gonorrhoea and this may have influenced our findings. Our study had a substantial proportion of missing incubation data (38.1%) because this data was retrospectively collected from clinical records. Due to missing incubation data for urethral gonorrhoea, many men with test results for oropharyngeal gonorrhoea were not included in our final analysis; however, we did not see differences between those who had incubation data and men who did not have incubation data. Third, we did not have data on who did and did not use saliva for masturbation. Last, we did not collect data on the use of antibacterial mouthwash, which may inhibit the growth of *N. gonorrhoeae* found in the oropharynx [28].

Our findings suggest that the incubation period for urethral gonorrhoea is not affected by whether men had concurrent oropharyngeal gonorrhoea. The data from our study, although yielding statistical non-significance, contribute to a growing body of research on the dynamics of gonorrhoea transmission, and calls for prospective research on the incubation period and transmission of gonorrhoea between anatomical sites, which may have implications for future prevention strategies. Understanding the transmission dynamic for the transmission of gonorrhoea is important for the development of accurate health messaging and interventions for gonorrhoea control in men.

## Data Availability

All data generated and analyzed during this study are included in the published article.
